# Radiomics Analyses to Predict Histopathology in Patients with Metastatic Testicular Germ Cell Tumors before Post-Chemotherapy Retroperitoneal Lymph Node Dissection

**DOI:** 10.3390/jimaging9100213

**Published:** 2023-10-07

**Authors:** Anna Scavuzzo, Giovanni Pasini, Elisabetta Crescio, Miguel Angel Jimenez-Rios, Pavel Figueroa-Rodriguez, Albert Comelli, Giorgio Russo, Ivan Calvo Vazquez, Sebastian Muruato Araiza, David Gomez Ortiz, Delia Perez Montiel, Alejandro Lopez Saavedra, Alessandro Stefano

**Affiliations:** 1 Department of Uro-Oncology, Instituto Nacional de Cancerologia, Universidad Autonoma de Mexico-UNAM, Mexico City 14080, Mexico; annasc80@gmail.com (A.S.);; 2 Institute of Molecular Bioimaging and Physiology, National Research Council (IBFM-CNR), 90015 Cefalù, Italy; giovanni.pasini@uniroma1.it (G.P.); giorgio.russo@ibfm.cnr.it (G.R.); 3 Department of Mechanical and Aerospace Engineering, Sapienza University of Rome, Eudossiana 18, 00184 Rome, Italy; 4 Science Department, Tecnológico de Monterrey, Mexico City 14080, Mexico; elisabetta.crescio@gmail.com; 5 Department of Biomedical Engineering, Instituto Nacional de Cancerologia, Universidad Autonoma de Mexico-UNAM, Mexico City 14080, Mexico; 6 Ri.MED Foundation, Via Bandiera 11, 90133 Palermo, Italy; acomelli@fondazionerimed.com; 7 Department of Pathology, Instituto Nacional de Cancerología, Mexico City 14080, Mexico; 8 Advanced Microscopy Applications Unit (ADMiRA), Instituto Nacional de Cancerología, Mexico City 14080, Mexico

**Keywords:** radiomics, metastatic non-seminomatous testicular germ cell tumors, computed tomography, histopathology

## Abstract

Background: The identification of histopathology in metastatic non-seminomatous testicular germ cell tumors (TGCT) before post-chemotherapy retroperitoneal lymph node dissection (PC-RPLND) holds significant potential to reduce treatment-related morbidity in young patients, addressing an important survivorship concern. Aim: To explore this possibility, we conducted a study investigating the role of computed tomography (CT) radiomics models that integrate clinical predictors, enabling personalized prediction of histopathology in metastatic non-seminomatous TGCT patients prior to PC-RPLND. In this retrospective study, we included a cohort of 122 patients. Methods: Using dedicated radiomics software, we segmented the targets and extracted quantitative features from the CT images. Subsequently, we employed feature selection techniques and developed radiomics-based machine learning models to predict histological subtypes. To ensure the robustness of our procedure, we implemented a 5-fold cross-validation approach. When evaluating the models’ performance, we measured metrics such as the area under the receiver operating characteristic curve (AUC), sensitivity, specificity, precision, and F-score. Result: Our radiomics model based on the Support Vector Machine achieved an optimal average AUC of 0.945. Conclusions: The presented CT-based radiomics model can potentially serve as a non-invasive tool to predict histopathological outcomes, differentiating among fibrosis/necrosis, teratoma, and viable tumor in metastatic non-seminomatous TGCT before PC-RPLND. It has the potential to be considered a promising tool to mitigate the risk of over- or under-treatment in young patients, although multi-center validation is critical to confirm the clinical utility of the proposed radiomics workflow.

## 1. Introduction

Germ cell cancer is a prevalent type of solid neoplasm that primarily affects young adult men within the age range of 18 to 44 years [1]. Among testicular tumors, testicular germ cell tumors (TGCT) are particularly noteworthy, and the incidence of this cancer has been on a steady rise in various developed countries over the past few decades. While advancements in diagnosis and treatment have brought this disease into focus, it remains unique among malignant diseases in terms of its limited number of identified risk factors. The precise reasons behind the increase in testicular cancer cases are still unknown, and our understanding of these risk factors remains relatively limited. Numerous studies have suggested potential links between exposure to various factors during adolescence and adulthood and the development of testicular cancer [2].

TGCT metastasis commonly occurs in the retroperitoneal lymph nodes located in the abdominal region behind the peritoneum [3]. These are the most frequent sites where TGCTs spread beyond the initial site of origin [4]. The combination of chemotherapy and surgery has resulted in a cure rate of >90%, and this result was achieved with meticulous management of retroperitoneal metastasis. The propensity for metastasis is a crucial aspect to consider in the diagnosis, staging, and treatment planning of patients with TGCT. As such, a comprehensive understanding of the behavior and patterns of metastasis in TGCTs can significantly impact patient outcomes and guide clinical management decisions [5]. Therefore, ongoing research efforts focus on improving our knowledge of the biological mechanisms driving TGCT metastasis to enhance early detection, prognosis, and therapeutic strategies for this group of young male patients. 

In the era of precision medicine, Artificial Intelligence (AI)-based image analysis emerges as a groundbreaking solution to tackle the challenges associated with traditional medicine [6]. This cutting-edge approach offers distinct advantages, including its non-invasive nature. By leveraging AI algorithms, radiomics focuses on analyzing texture features within a volume of interest (VOI) derived from medical images. These features serve as valuable indicators of tumor physiology and radiologic phenotype [7]. With its ability to extract meaningful information from images, AI-based image analysis holds great promise for enhancing diagnostic accuracy, personalized treatment planning, and patient care, driving us closer to a future of more precise and effective healthcare interventions.

In this paper, we aimed to investigate the potential of the radiomics model in predicting histopathology outcomes (i.e., fibrosis/necrosis, teratoma, and viable tumor) following post-chemotherapy retroperitoneal lymph node dissection (PC-RPLND) in metastatic non-seminomatous testicular cancer. This cancer presents a significant clinical challenge, and PC-RPLND is a crucial surgical intervention aimed at removing residual masses after systemic chemotherapy. The successful outcome of PC-RPLND greatly depends on the ability to accurately predict the histopathological characteristics of the residual masses, as it influences treatment decisions and patient prognosis. Through the use of radiomics, quantitative features from medical images are extracted and analyzed [8]. These features provide valuable insights into the underlying tumor biology and have shown potential for predicting treatment responses and patient outcomes across various cancer types. 

For these reasons, we conducted a retrospective analysis of preoperative imaging data, specifically computed tomography (CT) images, from patients who underwent PC-RPLND for metastatic non-seminomatous testicular cancer. Our principal aim was to identify the radiomics features that most strongly correlate with specific histopathological outcomes. We sought to assess their potential as non-invasive predictive biomarkers for guiding treatment decisions, stratifying patient risk, and monitoring therapeutic responses [9]. This investigation was conducted within the context of personalized and precision therapy for this particularly aggressive form of testicular cancer.

## 2. Materials and Methods

The residual masses of patients who underwent PC-RPLND for meta-static non-seminomatous testicular cancer were accurately delineated in CT images, and a wide range of radiomics features from the segmented regions were extracted, capturing information related to the tumor’s shape, intensity, texture, and spatial relationships. These radiomics features serve as quantitative biomarkers reflecting the tumor’s phenotypic characteristics, heterogeneity, and microenvironment. To establish the association between radiomics features and histopathology (i.e., fibrosis/necrosis, teratoma, and viable tumor), we correlated the extracted features with the corresponding surgical pathology results obtained from the PC-RPLND specimens. Therefore, we performed statistical analyses and developed predictive models using machine learning algorithms to optimize the classification performance. The following subsections provide a detailed description of the various parts of the adopted methodology.

### 2.1. Study Design

We enrolled patients with metastatic testicular germ cell tumors at diagnosis as candidates for retroperitoneal surgery to extirpate residual tumors after chemotherapy. We considered patients with seminoma and no seminoma tumors. Specifically, the collected dataset consisted of CT images of patients affected by metastatic TGCT who underwent PC-RPLND between January 2015 and December 2021. Electronic medical records of all consecutive TGCTs (*n* = 560) treated by the multidisciplinary uro-oncology team were retrospectively reviewed. 

**Figure 1:**
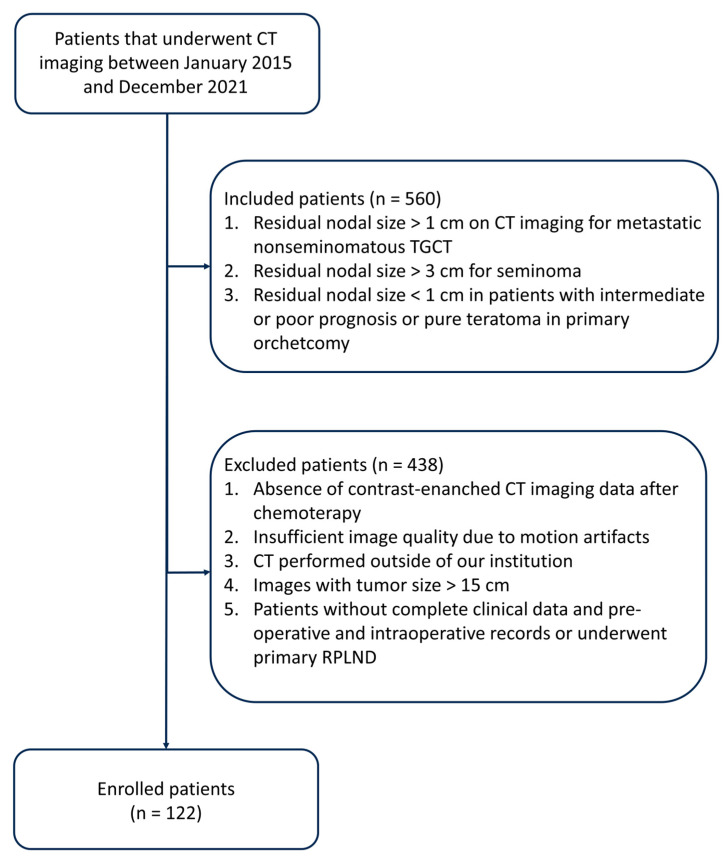
Diagram flowchart that summarizes the process of patient inclusion.

In this study, as illustrated in Figure 1, the inclusion criteria comprised the following:

(a) Residual nodal size exceeding 1 cm, as determined through transverse axial dimension on CT imaging, following frontline cisplatin-based chemotherapy for metastatic non-seminomatous TGCT.
(b) Residual nodal size exceeds 3 cm in cases of seminoma.
(c) Residual nodal size measuring less than 1 cm in patients exhibiting intermediate or poor prognosis or pure teratoma in the primary orchiectomy specimen.

The exclusion criteria encompassed the following:

(a) Lack of contrast-enhanced CT imaging data post-chemotherapy.
(b) Inadequate image quality is attributable to motion artifacts.
(c) CT scans were conducted at external institutions.
(d) Images displaying a tumor size exceeding 15 cm.
(e) Patients without comprehensive clinical data, pre-operative and intraoperative records, or patients who underwent primary RPLND.

For all patient examinations, a CT scanner from Siemens Healthineers was utilized, specifically the SOMATOM Definition Flash model. The acquired images were displayed in the axial plane, with a slice thickness ranging from 2.0 to 5.0 mm and an in-plane resolution varying between 0.62 × 0.62 mm and 0.86 × 0.86 mm. Standard CT configurations were employed for the thorax, abdomen, and pelvis, following conventional protocols.

All surgery is carried out by three surgeons with ten years of experience. All patients were treated with conventional open surgery, preferring a transabdominal approach via a midline laparotomy incision or minimally invasive retroperitoneal lymph node dissection. It was performed in all patients beyond the resection of residual mass on a unilateral or bilateral modified template, including all ipsilateral lymph nodes between the level of the renal vessels and the bifurcation of the common iliac artery. 

Finally, 122 patients with metastatic TGCT, underwent PC-RPLND were included in the radiomics analysis. Each patient was labeled according to the following histopathology: fibrosis/necrosis (57 observations), teratoma (48 observations), viable tumor (17 observations).

This study complies with the Declaration of Helsinki, and local ethics committee approval was obtained (Instituto Nacional de Cancerología of Mexico, n. 2020/0123).

### 2.2. Segmentation

**Figure 2:**
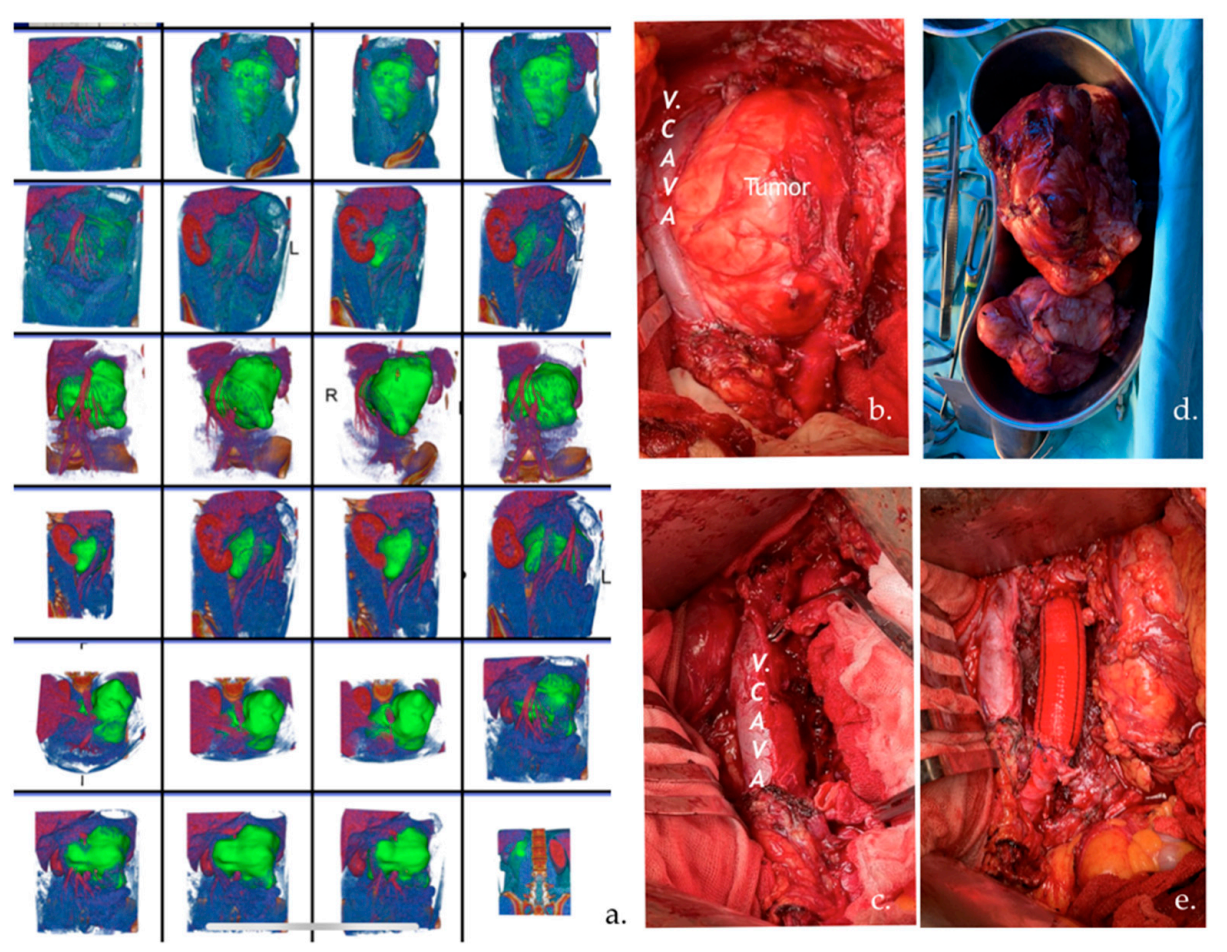
(**a**) CT images with 3D segmentation of a 30-year-old patient with an enlarged left pericaval lymph node (LN) after chemotherapy. The patient underwent PC-RPLND. The volume of LN was 15 cm in diameter. (**b**,**c**) Pictures of intraoperative retroperitoneal surgery of the tumor that displaced the v. cava and infiltrated the aorta. (**d**) The tumor was totally resected. (**e**) Aortic vascular prosthesis.

Image segmentation was performed semi-automatically using the 3D Slicer open-source software platform (version 4.8; www.slicer.orgwww.slicer.org) (accessed on 28 August 2023) by an experienced biomedical engineer and uro-oncologist with 10 years of experience. After loading the Digital Imaging and Communications in Medicine (DICOM) files, we used images of CT in the arterial and portal-venous contrast phases to create 3D lymph node segmentation with manual corrections. Specifically, we used MONAILabel (https://monai.io/) (accessed on 1 April 2023) and R-Vessel-X (http://tgi.ip.uca.fr/r-vessel-x) (accessed on 1 April 2023) plugins to create and edit segmentations. Figure 2 presents an example of a 3D segmentation and the corresponding resected tumor.

### 2.3. Radiomics Feature Extraction

A wide range of radiomics features were extracted from CT images and analyzed to uncover hidden information related to the investigated disease. These quantitative features include the texture, shape, and intensity characteristics of the target, in our case the residual masses of patients who underwent PC-RPLND for metastatic non-seminomatous testicular cancer. By systematically analyzing these features, radiomics aims to aid in disease diagnosis, prognosis, treatment planning, and therapeutic response assessment, ultimately contributing to personalized and more effective healthcare decisions. Specifically, in our study, PyRadiomics was used to extract 851 features belonging to the original images (107) and the images pre-processed through wavelet decomposition (744), which was performed by configuring the Filter Module of PyRadiomics. Moreover, all the features belonged to the Shape class (14 features), the First Order Statistics (162 features) class, and the Texture class, which can be further divided into 5 feature classes: the gray level co-occurrence matrix (GLCM) (216 features), the gray level run length matrix (GLRLM) (144 features), the gray level size zone matrix (GLSZM) (144 features), the neighboring gray tone difference matrix (NGTDM) (45 features), and the gray level dependence matrix (GLDM) (126 features). PyRadiomics parameters used for the feature extraction process were:

- diagnostics_Configuration_Settings.additionalInfo (True)
- Configuration_Settings.binWidth (25.0)
- Configuration_Settings.distancesforce2Ddimension (0)
- Configuration_Settings.interpolator (sitkBSpline).label (1.0)
- minimumROIDimensions (2)
- minimumROISize (null)
- normalize (false)
- diagnostics_Configuration_Settings.normalizeScale (1)
- diagnostics_Configuration_Settings.padDistance (5)
- diagnostics_Configuration_Settings.preCrop (false)
- diagnostics_Configuration_Settings.removeOutliers (null)
- diagnostics_Configuration_Settings.resampledPixelSpacing (null)
- diagnostics_Configuration_Settings.resegmentRange (null)
- diagnostics_Configuration_Settings.symmetricalGLCM (true)

### 2.4. Machine Learning Pipeline

**Figure 3:**
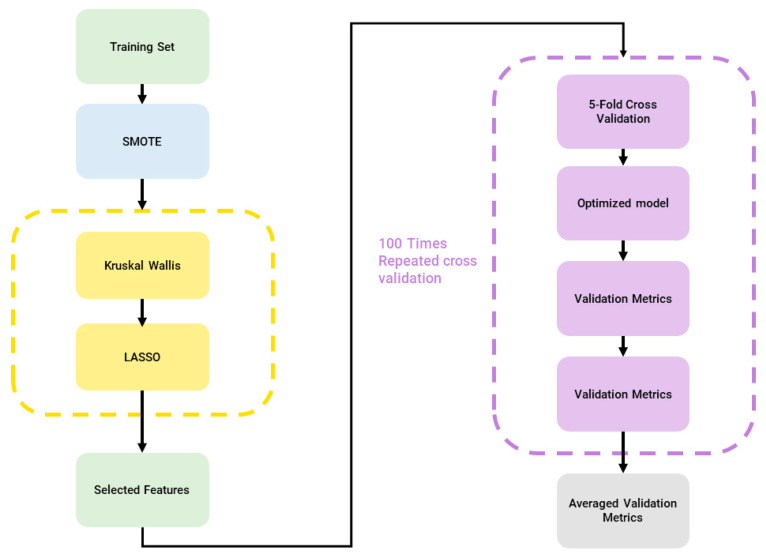
Machine Learning Pipeline, blue: dataset balancing, yellow: feature selection, purple: classification.

Firstly, clinical features and radiomics features were combined to obtain the final feature dataset (855 features) before sending it as input to the machine learning pipeline using a modified version of matRadiomics [10], to handle multiple outcomes, namely i. fibrosis/necrosis, ii. teratoma, and iii. viable tumor. The adopted pipeline consists of the following: (i) feature normalization through min-max normalization; (ii) Synthetic Minority Oversampling Technique (SMOTE) [11] to oversample the minority classes and balance the dataset; (iii) Kruskal–Wallis analysis followed by the least absolute shrinkage and selection operator (LASSO) for feature selection; (iv) model training; (v) model validation; and hyperparameter optimization. The machine learning pipeline is shown in Figure 3.

#### 2.4.1. Minority Classes Oversampling

Since the dataset was strongly unbalanced, we used SMOTE [11] to oversample the minority classes. In fact, when the distribution of data among classes is uneven in classification, the learning function is dominated by the features of the majority classes, resulting in a high misclassification rate for minority samples. Therefore, synthetic samples were created for the teratoma and tumor viable classes, thus increasing their amount from 48 to 57 and from 17 to 57, respectively, and the total number of cases from 122 to 171. 

#### 2.4.2. Feature Reduction and Selection

Given that the combined dataset comprises high-dimensional data, encompassing a substantial number of radiomics features (855), far exceeding the number of cases (171), it was imperative to employ feature reduction and selection techniques to streamline the model in terms of either training efficiency or model explainability. To achieve this, we employed Kruskal–Wallis to retain features with *p*-values below predefined thresholds and leveraged LASSO, a widely recognized radiomics feature selection algorithm, to identify the most important features. Consequently, we established three incremental thresholds (*p*-value < 0.005, *p*-value < 0.01, and *p*-value < 0.05) and adopted the following strategy for transitioning between these thresholds:

- In the event that none of the features meet the first threshold (*p* < 0.005), we elevated the *p*-value threshold to the second level;
- If the second threshold remains unmet, we escalate the *p*-value to the third threshold;
- If none of the features satisfied the third threshold, all the features were given as input to the LASSO. The optimal lambda value was determined through a rigorous 10-fold cross-validation process.

#### 2.4.3. Classification

We employed a robust validation approach by conducting 100 repeated iterations of stratified 5-fold cross-validation for model validation. This process also included the fine-tuning of hyperparameters [12]. To expedite the hyperparameter tuning process, we opted for Bayesian optimization. Performance metrics were based on the average of the 100 repetitions of the cross-validation procedure. Finally, six machine learning models, namely Discriminant Analysis Classifier (DC) [13], tree [14], K-nearest neighbors (KNN) [15], support vector machines (SVM) [16], Naïve Bayes (NB) [17], and ensemble [18], were trained, validated, and optimized.

#### 2.4.4. Statistical Analyses

For each trained model, we calculated several performance metrics, including:

- Accuracy ((TP + TN)/(TP + TN + FP + FN))
- True Positive Rate (TPR) or sensitivity (TP/(TP + FN))
- True Negative Rate (TNR) or specificity (TN/(TN + FP))
- Positive Predicted Value (PPV) or precision (TP/(TP + FP))
- The area under the receiver operating characteristic curve (AUC)
- F-score

where TP (true positive), TN (true negative), FP (false positive), and FN (false negative). The metrics TPR, TNR, PPV, AUC, and F-score were averaged across the three classes for each classifier. For instance, the average AUC (AUCav) was computed as the sum of the AUC values for each class (AUCclass1-SVM + AUCclass2-SVM + AUCclass3-SVM) divided by 3.

## 3. Results

### 3.1. Clinical Data

Clinical data are shown in Table 1: age, clinical stage at diagnosis, prognostic group according to International Germ Cell Cancer Collaborative Group (IGCCCG) classification, serum markers at diagnosis, primary histopathology, serum markers before PC-RPLND, type of PC-RPLND (standard, salvage, desperation, and redo-surgery), side of orchiectomy (left, right, bilateral, extragonadal, deferred), damage to organs, vascular damage. PC-RPLND standard refers to surgery after first-line chemotherapy and negative serum markers; PC-RPLND salvage after more lines of chemotherapy and negative serum markers; desperation RPLND applies to patients with persistently elevated or increasing serum tumor markers after primary inductive chemotherapy or after salvage chemotherapy; and redo PC-RPLND in cases with recurrent or persistent disease after surgery. We included age, side of orchiectomy, damage to organs, and vascular damage as clinical features in the radiomics analyses.

### 3.2. Feature Reduction and Selection

After feature extraction, since the combined dataset of extracted features and clinical features was high-dimensional (855 features), a feature selection pipeline was adopted to analyze these features, as reported in Section 2.4.2. The feature reduction (i.e., Kruskal–Wallis analysis) and selection (i.e., LASSO) processes reduced the total number of combined radiomics-clinical features (851 features extracted from the CT images and 4 clinical features) to the most predictive ones, thus producing a subset of 30 features. Only 3 features belonged to the original images, namely, original_firstorder_Median, original_firstorder_90Percentile, and original_glszm_LargeAreaEmphasis, while the rest all belonged to the wavelet decomposed images. No clinical features were selected as the most predictive ones. In Table 2, we report the 30 selected features associated with the *p*-value derived from the Kruskal–Wallis analysis. The lower the *p*-value, the more statistically significant the difference in feature values between groups (fibrosis/necrosis, teratoma, tumor viable) for a specific feature. As shown in Table 2, only First Order and Texture features were included in the subset of selected features, while Shape features were not present. For the features whose *p*-value was much lower than 0.005 (*p*-values whose order of magnitude ranged from 10^−12^ to 10^−4^, threshold = 5 × 10^−3^), we used the “<<0.005” notation. As shown in Table 3, which reports the number of selected features based on image type and class, 12 features belonged to the First Order Statistics class, 2 based on the original images and 10 on the wavelet decomposed images, and 18 features belonged to the Texture feature class, 1 based on the original images and 17 on the wavelet decomposed images. 

### 3.3. Classification

**Figure 4:**
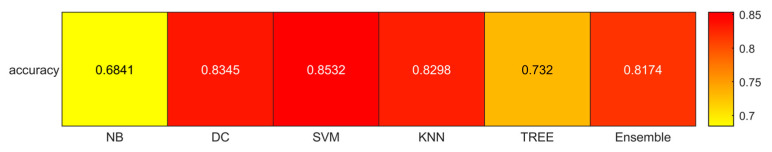
Accuracies for the six classifiers (NB, DC, SVM, KNN, TREE, ENSEMBLE).

**Figure 5:**
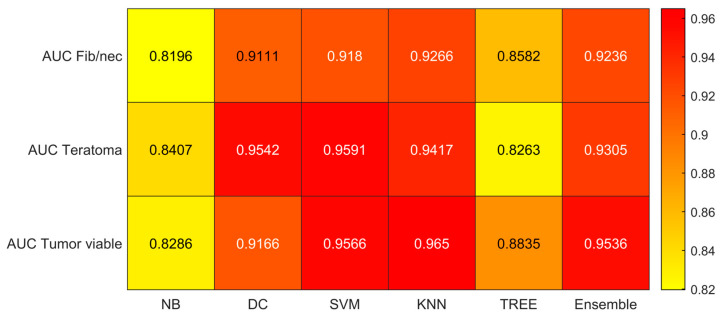
AUC values for the six classifiers (NB, DC, SVM, KNN, TREE, ENSEMBLE) and for each case (Fib/nec: fibrosis/necrosis, Teratoma, Tumor Viable).

**Figure 6:**
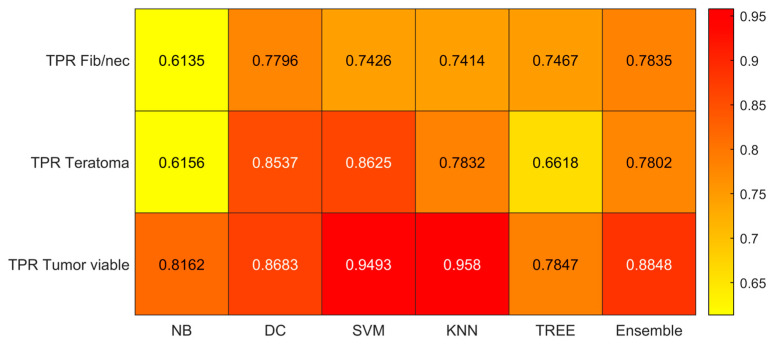
Sensitivity (TPR) values for the six classifiers (NB, DC, SVM, KNN, TREE, ENSEMBLE) and for each case (Fib/nec: fibrosis/necrosis, Teratoma, Tumor Viable).

**Figure 7:**
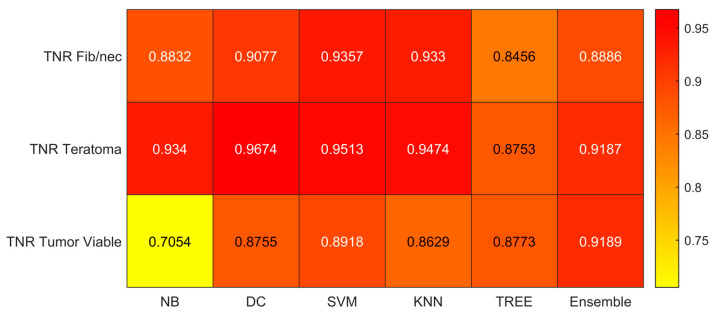
Specificity (TNR) values for the six classifiers (NB, DC, SVM, KNN, TREE, ENSEMBLE) and for each case (Fib/nec: fibrosis/necrosis, Teratoma, Tumor Viable).

**Figure 8:**
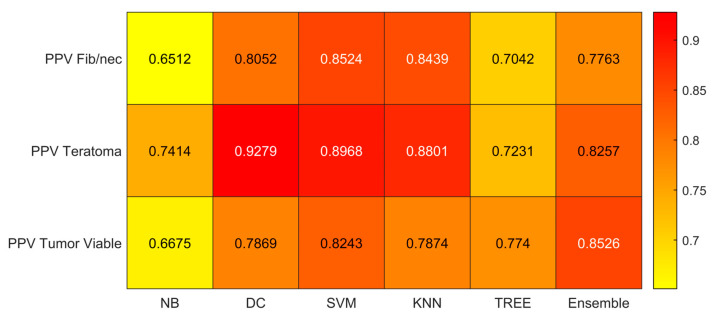
Precision (PPV) values for the six classifiers (NB, DC, SVM, KNN, TREE, ENSEMBLE) and for each case (Fib/nec: fibrosis/necrosis, Teratoma, Tumor Viable).

**Figure 9:**
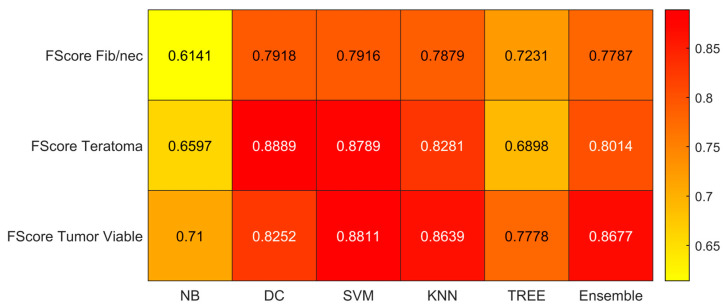
F-score values for the six classifiers (NB, DC, SVM, KNN, TREE, ENSEMBLE) and for each case (Fib/nec: fibrosis/necrosis, Teratoma, Tumor Viable).

We reported the accuracy, AUC, sensitivity (TPR), specificity (TNR), precision (PPV), and f-score averaged on the 100 times repeated 5-fold cross-validation for the six different classifiers (DC, KNN, SVM, Naïve Bayes, Tree, and Ensemble) from Figure 4, Figure 5, Figure 6, Figure 7, Figure 8 and Figure 9.

As reported in Figure 4, which shows the accuracies obtained by each classifier, the Support Vector Machines (SVM) obtained the highest accuracy (0.8532). Since we performed a multiclass classification (3 classes), results related to the AUC (Figure 5), TPR or sensitivity (Figure 6), TNR or specificity (Figure 7), PPV or precision (Figure 8), and F-score (Figure 9) are given, divided by class (Fibrosis/Necrosis, Teratoma, Tumor Viable), placed along the rows of the figures. Therefore, given a row, the corresponding class was considered the positive label, while the remaining classes were the negative labels. In such a way, each classifier measures the AUC, TPR, TNR, PPV, and f-score of one class (positive) against the remaining classes (negative). For each classifier and metric, averaging the results obtained for each class leads to the classifier global metric. As reported in Figure 5, the highest value of AUC (0.965) is reached when the KNN classifier is adopted to distinguish the Tumor Viable class from the other two classes, but in general, the SVM classifier reached the highest average AUC (0.945). 

**Figure 10:**
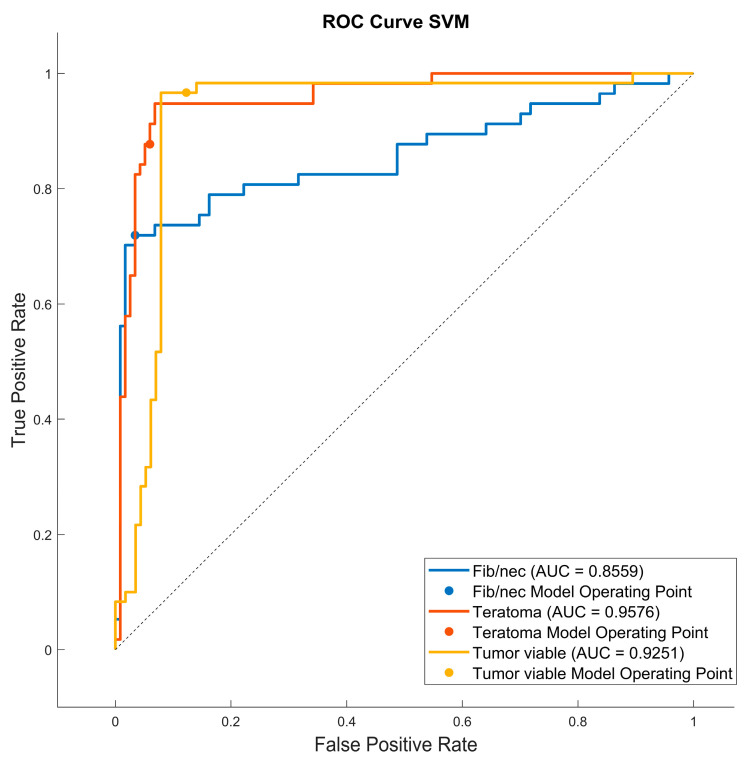
Roc curves for the SVM classifier for each case (Fib/nec: fibrosis/necrosis, Teratoma, Tumor Viable).

As reported in Figure 6, the highest value of TPR (0.958) is reached when the KNN classifier is adopted to distinguish the Tumor Viable class from the other two classes, but in general, the SVM classifier reached the highest average TPR (0.851). As reported in Figure 7, the highest value of TNR (0.967) is reached when DC is adopted to distinguish the Teratoma class from the other two classes, but in general, the SVM classifier reached the highest TNR (0.926). As reported in Figure 8, the highest value of PPV (0.928) is reached when DC is adopted to distinguish the Teratoma class from the other two classes, but in general, the SVM classifier reached the highest average PPV (0.858). As reported in Figure 9, the highest value of f-score (0.889) is reached when DC is adopted to distinguish the Teratoma class from the other two classes, but in general, the SVM classifier reached the highest f-score (0.851). All the values are rounded to three decimal places. Moreover, we reported an example of receiver operating characteristic (ROC) curves obtained in one repetition of the cross-validation procedure for the most performant classifier, namely the SVM, in Figure 10. Each curve (three in total) describes the ability of the classifier to distinguish one class from the remaining classes. The larger the area under the curve, the better the result.

## 4. Discussion

In the proposed study, we developed a radiomics model for the individualized preoperative prediction of histopathology in metastatic non-seminomatous TGCT before PC-RPLND. Residual nodal masses are observed in around 40% of patients following chemotherapy, and surgical intervention is recommended when post-chemotherapy axial nodal measurements exceed 1 cm. The primary goal of surgery is to excise residual mature teratoma or viable tumors, which are present in approximately 30% to 40% and 5% to 10% of surgical specimens, respectively. Consequently, approximately 50% of patients have fibrosis/necrotic tissue [19]. These findings were confirmed in our study cohort, where we observed 57 fibrosis/necrosis (47% of the entire cohort), 48 teratoma (39%), and 17 viable tumors (14%). 

To date, the most commonly employed algorithm for histopathology identification relies on six clinical variables: prechemotherapy tumor markers [alpha-fetoprotein, beta-human chorionic gonadotropin, lactate dehydrogenase], residual mass size, percentage of mass shrinkage, and the presence of teratoma elements in the orchiectomy specimen [20]. Although it has demonstrated a high level of discriminative accuracy, with AUC values ranging from 0.77 to 0.84, this model has not been widely embraced in clinical practice due to its inherent complexity [21]. In addition, according to the European Association of Urology 2023 guidelines, contrast-enhanced CT stands out as the most sensitive method for assessing the thorax, abdomen, and pelvis in the staging of testicular cancer. The recommendation advocates the use of contrast-enhanced CT for staging in all patients prior to orchidectomy; however, it may be postponed until histopathological confirmation of malignancy is obtained. 

For these reasons, the main result of our radiomics study can be summarized as follows: Using an adapted version of matRadiomics [10], starting from 4 clinical parameters that can be easily collected together with 851 CT imaging features, we developed a predictive model based on 30 radiomics features after the selection and reduction process. The proposed radiomics model, implemented using SVM, demonstrated optimal accuracy (85%) in predicting histopathological outcomes, specifically differentiating between fibrosis/necrosis, teratoma, and viable tumors. To ensure the reliability of our approach, we assessed its robustness through 100 iterations of 5-fold cross-validation. In particular, as detailed in Section 3, the results indicate that the performance of various classifiers varies based on the specific class they aim to distinguish from the other two classes. However, when considering overall performance, SVM consistently outperforms the others. SVM achieves the highest overall average AUC (0.945), the highest overall average TPR (0.851), the highest overall average TNR (0.926), the highest overall average PPV (0.858), and the highest overall average F-score (0.851). Conversely, Naïve-Bayes demonstrates the least favorable performance.

In the literature the results of usefulness of the radiomics in testicular cancer in primary and post-chemotherapy settings have been controversial. Similarly to our study, Baessler et al.

demonstrated that a SVM classifier, utilizing radiomics data from CT scans, had the capability to predict lymph node histopathology following lymph node dissection in patients with TGCT who had undergone chemotherapy [22]. This single-center retrospective study included 81 patients with a total of 204 lesions and achieved 81% accuracy in classifying them into “benign” (necrosis/fibrosis) or “malignant” (viable tumor/teratoma), unlike our study, where a 3-outcome classification was performed (fibrosis/necrosis, teratoma, and viable tumor). In addition, they did not include clinical variables in the proposed radiomics approach, even though, in our case, clinical features were not identified as the most predictive ones by the feature selection and reduction algorithms. Furthermore, they partitioned this study cohort, which was of moderate size, into three subgroups. The test group comprised merely 19 patients, leading to an overall decrease in statistical significance. To address this limitation, we adopted a cross-validation methodology based on recurrent data partitioning, serving the dual purpose of guarding against overfitting and yielding precise model coefficient estimates [23]. Venishetty et al., using Pyradiomics in 45 patients undergoing PC- RPLND, failed to prove that radiomics analysis can predict pathology after retroperitoneal surgery [24]. In another retrospective study conducted at a single center [25], involving 77 patients with metastatic TGCT and a total of 102 lesions, the accuracy of radiomics to identify germ cell tumor vs teratoma vs fibrosis was 72 ± 2.2% (area under the curve [AUC], 0.74 ± 0.028); sensitivity was 56.2 ± 15.0%, and specificity was 81.9 ± 9.0%. Upon incorporating clinical variables such as the presence of teratoma in the primary tumor, pre-chemotherapy tumor marker levels, and pre- and post-chemotherapy mass size into the radiomics signature, the optimal classifier was determined. This classifier demonstrated superior performance when applied exclusively to axial masses with a diameter less than 2 cm, achieving an accuracy of 88.2% and an AUC of 0.80. They implemented a nested 10-fold cross-validation protocol to ascertain the accuracy of the classifier. Unlike this study, we aimed to discriminate among three distinct tissues, not restricting our focus to masses with diameters smaller than 2 cm. Nevertheless, we still achieved high levels of accuracy; our results argue in favor of the use of radiomics to predict histopathology through a machine learning pipeline based on matRadiomics. Notwithstanding that PC-RPLND for residual tumors is pivotal in the management of TGCT, a complicated dispute continues to avoid surgery cautiously. So, the use of radiomics could be included in the current guidelines [26,27,28] to distinguish which patients should be treated by surgery or kept under surveillance. However, radiomics is a relatively recent field and is evolving rapidly. It does not constitute a perfect solution or replace clinical management. Radiomic features can provide information about the tumor and support clinical decisions. [29,30]. Radiomics in conjunction with standard clinical predictors might help you decide if to keep patients under surveillance in case of residual necrosis or perform retroperitoneal surgery promptly so as to prevent undertreatment or overtreatment. PC-RPLN, although necessary, remains a surgery with a broad complication rate [31].

Finally, it is essential to acknowledge the potential limitations of the present radiomics study. By addressing the challenges mentioned below, we hope to pave the way for personalized and precise therapeutic approaches in the management of this aggressive form of testicular cancer.

First, since this study is retrospective in nature and employs a relatively small cohort, it is critical to highlight the potential presence of inherent selection bias.

Second, classes were highly unbalanced (57 fibrosis/necrosis, 48 teratoma, and 17 viable tumors). To overcome this limitation, we used SMOTE [11] to oversample the minority classes and balance the dataset. In addition, in contrast to previous radiomics studies focused on LN metastasis, which primarily extracted features from the largest cross-sectional area, our study conducted a comprehensive whole-lesion analysis by encompassing all available CT slices. This approach yielded a wealth of information concerning tumor heterogeneity. 

Third, our case was a single-institution study without an external validation cohort. Therefore, prospective, multicenter validation is essential to gathering more robust evidence for clinical application. Nonetheless, considering our approach based on fold cross-validation, we hold confidence that our integrated prediction model exhibits generalizability. Future studies should aim to validate our trained model in prospective research settings [32].

Fourth, the use of an operator-independent segmentation system is mandatory in the study of radiomics [33]. Although we used semi-automatic segmentation, this may suffer from inter-observer variability. This variability provides less precise and mostly irreproducible results since the radiomics signature is strongly influenced by the VOI designed to identify the tumor.

Finally, this is a radiomics study based on CT alone; if multiple modalities are combined, such as CT, PET, and MRI, the resulting feature set could increase the ability to predict histopathology in TGCT patients [34]. 

## 5. Conclusions

The presented CT-based radiomics model may potentially serve as a non-invasive tool for predicting histopathology differentiation among fibrosis/necrosis, teratoma, and viable tumor in metastatic non-seminomatous TGCT before PC-RPLND. It has the potential to be considered a useful tool in mitigating the risk of over- or under-treatment. In other words, the proposed model could help in the future to predict residual histology in the retroperitoneum after chemotherapy. We observed the propensity of the model to anticipate teratoma histology. The future objective is to predict the presence of fibrosis to avoid surgery on residual tumors smaller than one centimeter. Radiomics and the application of artificial intelligence in TGCT are fascinating areas that should be considered to improve the quality of life of patients suffering from this disease. 

## Figures and Tables

**Table 1:** Clinical data.

	Fibrosis/Necrosis	Teratoma	Viable Tumor
Patient age at diagnosis (years)	26.03 ± 6.46	23.94 ± 5.95	28.81 ± 11.66
Clinical stage (initial)			
I	2/57 (3.51%)	7/48 (14.6%)	9/17 (52.9%)
II	20/57 (35.1%)	16/48 (33.3%)	6/17 (35.3%)
III	35/57 (61.4%)	25/48 (52.1%)	2/17 (11.7%)
IGCCCG (initial)			
Good	14/57 (24.6%)	15/48 (31.2%)	5/17 (29.4%)
Intermediate	26/57 (45.6%)	19/48 (39.6%)	7/17 (41.2%)
Poor	13/57 (22.8%)	12/48 (25.0%)	5/17 (29.4%)
Missing information	4/57 (7.0%)	2/48 (4.2%)	0/17 (0%)
Serum Marker initial			
AFP	2278 ± 7246	3639 ± 8481	5401 ± 9912
hCG	3596 ± 12,899	13,436 ± 40,984	925 ± 1331
LDH	1334 ± 2202	475 ± 384	546 ± 431
Primary histopathology			
Seminoma	13/57 (22.8%)	1/48 (0.021%)	1/17 (0.059%)
Non-seminoma	44/57 (77.2%)	47/48 (97.9%)	16/17 (94.12%)
Containing teratoma	1/57 (0.017%)	2/48 (0.042%)	6/17 (35.3%)
Without teratoma	56/57 (98.2%)	46/48 (95.8%)	11/17 (64.7%)
Type of pcRPLND			
Primary	2/57 (3.5%)	0/48 (0%)	2/17 (11.8%)
Standard	8/57 (14.0%)	10/48 (20.8%)	0/17 (0%)
Salvation	41/57 (71.9%)	28/48 (58.3%)	6/17 (35.3%)
Desperation	5/57 (8.8%)	9/48 (18.8%)	5/17 (29.4%)
Redo	1/57 (1.8%)	1/48 (2.1%)	4/17 (23.5%)
Serum marker prior pcRPLN			
AFP	4.0 ± 3.5	6.6 ± 11.8	56.4 ± 91.2
hCG	3.1 ± 18.5	5.3 ± 30.0	21.7 ± 83.8
LDH	195 ± 105	247.7 ± 170.1	631.3 ± 1461.3
Side of orchiectomy			
Left	31/57 (54.4%)	30/48 (62.5%)	7/17 (41.2%)
Right	19/57 (33.3%)	16/48 (33.3%)	9/17 (52.9%)
Bilateral	2/57 (3.5%)	1/48 (2.1%)	1/17 (5.9%)
Extragonadal	1/57 (1.8%)	1/48 (2.1%)	0/17 (0%)
Deferred	4/57 (7%)	0/48 (0%)	0/17 (0%)
Damage to organs			
Yes	4/57 (7%)	2/48 (4.2%)	1/17 (5.9%)
No	53/57 (93%)	46/48 (95.8%)	16/17 (94.1%)
Vascular damage			
Yes	4/57 (7%)	7/48 (14.6%)	2/17 (11.8%)
No	53/57 (93%)	41/48 (85.4%)	15/17 (88.2%)
Volume (cm^3^)	65.8 ± 132.4	505.4 ± 744.6	1156.6 ± 1689.3

**Table 2:** Subset of selected features and their *p*-value obtained through the Kruskal–Wallis analysis.

Selected Features	*p*-Value
original_firstorder_Median	<<0.005
wavelet_LLH_glcm_MCC	<<0.005
original_firstorder_90Percentile	<<0.005
wavelet_LLL_glcm_Idmn	<<0.005
wavelet_LLL_firstorder_RootMeanSquared	<<0.005
wavelet_HHH_glszm_LargeAreaEmphasis	<<0.005
wavelet_HLH_glszm_SmallAreaEmphasis	<<0.005
wavelet_HHH_glszm_LargeAreaHighGrayLevelEmphasis	<<0.005
wavelet_HHH_firstorder_Median	<<0.005
wavelet_HLH_glrlm_RunLengthNonUniformityNormalized	<<0.005
wavelet_LHL_glrlm_RunLengthNonUniformityNormalized	<<0.005
wavelet_LLH_glcm_InverseVariance	<<0.005
wavelet_LLH_gldm_SmallDependenceEmphasis	<<0.005
wavelet_LLH_glszm_ZoneVariance	<<0.005
wavelet_LLL_firstorder_10Percentile	<<0.005
wavelet_HLH_glszm_LargeAreaEmphasis	<<0.005
wavelet_HHL_firstorder_Minimum	<<0.005
wavelet_LHL_glszm_SmallAreaHighGrayLevelEmphasis	<<0.005
wavelet_LHH_firstorder_Range	<<0.005
wavelet_LHH_glrlm_LongRunHighGrayLevelEmphasis	0.0011
wavelet_LLH_firstorder_Minimum	0.0017
wavelet_HHL_glszm_SizeZoneNonUniformityNormalized	0.0018
wavelet_HHL_glrlm_RunLengthNonUniformityNormalized	0.0021
wavelet_LLH_firstorder_Kurtosis	0.0022
wavelet_LHH_ngtdm_Complexity	0.0028
wavelet_HLH_glcm_MCC	0.0040
original_glszm_LargeAreaEmphasis	0.0041
wavelet_LHH_gldm_DependenceNonUniformity	0.0042
wavelet_HLL_firstorder_10Percentile	0.0045
wavelet_HHL_gldm_HighGrayLevelEmphasis	0.0048

**Table 3:** Number of selected features based on Image Type and Class.

Image Type	Class	Number of Features
Original	Shape	0
Original	First Order	2
Original	Texture	1
Wavelet	Shape	0
Wavelet	First Order	10
Wavelet	Texture	17

## Data Availability

Not applicable.
